# Genome Sequencing of Three Pathogenic Fungi Provides Insights into the Evolution and Pathogenic Mechanisms of the Cobweb Disease on Cultivated Mushrooms

**DOI:** 10.3390/foods13172779

**Published:** 2024-08-30

**Authors:** Yufei Lan, Qianqian Cong, Qingwei Yu, Lin Liu, Xiao Cui, Xiumei Li, Qiao Wang, Shuting Yang, Hao Yu, Yi Kong

**Affiliations:** 1 Institute of Edible Fungi, Tai’an Academy of Agricultural Sciences, Tai’an 271000, China; lanyufei526@163.com (Y.L.); cong.qianqian@163.com (Q.C.); zsztyqw@126.com (Q.Y.); cuixiao916@163.com (X.C.); tanlixiumei@163.com (X.L.); 18815380509@163.com (Q.W.); 2 Shandong Provincial Key Laboratory of Applied Mycology, School of Life Sciences, Qingdao Agricultural University, Qingdao 266109, China; liulin@qau.edu.cn (L.L.); yangshuting0701@163.com (S.Y.)

**Keywords:** edible mushrooms, Hypocreaceae, comparative genome, pathogenesis

## Abstract

Fungal diseases not only reduce the yield of edible mushrooms but also pose potential threats to the preservation and quality of harvested mushrooms. Cobweb disease, caused primarily by fungal pathogens from the Hypocreaceae family, is one of the most significant diseases affecting edible mushrooms. Deciphering the genomes of these pathogens will help unravel the molecular basis of their evolution and identify genes responsible for pathogenicity. Here, we present high-quality genome sequences of three cobweb disease fungi: *Hypomyces aurantius* Cb-Fv, *Cladobotryum mycophilum* CB-Ab, and *Cladobotryum protrusum* CB-Mi, isolated from *Flammulina velutipes*, *Agaricus bisporus*, and *Morchella importuna*, respectively. The assembled genomes of *H. aurantius*, *C. mycophilum*, and *C. protrusum* are 33.19 Mb, 39.83 Mb, and 38.10 Mb, respectively. This is the first report of the genome of *H. aurantius*. Phylogenetic analysis revealed that cobweb disease pathogens are closely related and diverged approximately 17.51 million years ago. CAZymes (mainly chitinases, glucan endo-1,3-beta-glucosidases, and secondary metabolite synthases), proteases, KP3 killer proteins, lipases, and hydrophobins were found to be conserved and strongly associated with pathogenicity, virulence, and adaptation in the three cobweb pathogens. This study provides insights into the genome structure, genome organization, and pathogenicity of these three cobweb disease fungi, which will be a valuable resource for comparative genomics studies of cobweb pathogens and will help control this disease, thereby enhancing mushroom quality.

## 1. Introduction

With the development of modern society and the increasing pursuit of healthful foods, edible mushrooms have become increasingly popular [1,2]. The total production of mushrooms and truffles worldwide reached 48.34 million metric tons in 2022 according to the data from the Statista website (statista.com). Most edible fungi are produced through commercially artificial cultivation [2,3,4]. Consequently, the outbreaks of diseases caused by fungal pathogens have continuously increased in recent years [5,6,7]. Mycopathogens cause heavy losses in commercial mushroom cultivation worldwide, such as cobweb disease, dry bubble disease, wet bubble disease, and green mold disease [6]. Cobweb disease is a significant limiting factor in edible mushroom production [8,9]. The main symptom of cobweb disease is a cobweb-like growth of fungal mycelia over the mushroom surface. The fruit body shows discoloration and rot and eventually becomes unsellable. The spores of pathogenic fungi are easily dispersed through air conditioning systems or by the action of watering [9]. If left untreated, the disease will spread throughout the mushroom crop through airborne spore dispersal [10]. In recent years, cobweb disease has been widespread and has caused serious losses in Europe, America, Africa, and Asia [9]. In the mid-1990s, cobweb disease emerged as the most serious disease affecting mushroom cultivation in the UK and Ireland. The prevalence of cobweb disease in *Agaricus bisporus* production has been documented at 33% in Turkey and 32% in Spain. Production losses can reach as high as 40% [9]. The earliest case of edible mushroom cobweb disease was reported in *A. bisporus* [11]. Subsequently, there has been an increasing number of reports describing the infection of other mushrooms by cobweb disease. Due to the inability to thoroughly sterilize the soil, mushrooms requiring casing soil in cultivation, such as *Coprinus comatus*, *Morchella importuna*, and *Oudemansiella raphanipes*, are particularly susceptible to cobweb disease [12,13,14]. A strain causing cobweb disease was isolated by Xu et al. and was from the most widely consumed mushroom *Lentinula edodes* [15]. Cobweb disease has also been observed in the widely cultivated *Pleurotus* spp., such as *P. ostreatus*, *P. pulmonarius*, and *P. eryngii* [16,17]. Industrially cultivated edible mushrooms, such as *Flammulina velutipes* and *Hypsizygus marmoreus*, could also be infected by cobweb mycopathogens [18,19]. Cobweb disease has become a serious disease in mushroom crops. Therefore, an in-depth study of the pathogenic fungi causing cobweb disease is imperative to better control this disease.

Strains from *Hypomyces* and *Cladobotryum* genera were the most commonly reported pathogenic fungi causing cobweb disease [8]. Both genera belong to the family Hypocreaceae, Ascomycota Fungi, indicating that the fungi responsible for cobweb disease are closely related, and that the pathogenic traits are inherited from ancestors. *Cladobotryum mycophilum*, the anamorph of *Hypomyces odoratus*, is currently the most cited causal agent of cobweb disease. It has been described as a pathogen of various edible mushrooms, including *A. bisporus*, *P. eryngii*, *G. lucidum*, and *Morchella sextelata* [13,20,21,22]. *Cladobotryum dendroides*, *Cladobotryum protrusum*, and *Cladobotryum varium* were also reported as infecting various species of edible mushrooms, such as *C. comatus*, *M. importuna*, *L. edodes*, *O. raphanipes*, *F. velutipes*, and *H. marmoreus* [14,15,19,23,24]. *Hypomyces*, another genus of pathogenic fungi causing cobweb disease, has been discovered infecting *F. velutipes*, *A. bisporus*, *Auricularia cornea*, and *Auricularia heimuer* with the corresponding pathogens *Hypomyces aurantius*, *Hypomyces mycophilus*, *Hypomyces cornea*, and *Hypomyces rosellus* [11,18,25,26,27]. Over the past years, studies on edible fungal pathogens have mainly focused on the identification of pathogens and fungicide resistance, while research on the pathogenic mechanism of the pathogen is rare. Genome sequencing and analysis have facilitated the study of the characteristics of edible fungal pathogens and the pathogenicity-related genes, laying a theoretical foundation and indicating the direction for the further systematic study of pathogenic mechanisms [28,29,30].

Recently, the genomes of a few parasitic fungi that cause dry bubble, wet bubble, and green mold diseases in edible mushrooms have been released [31,32,33]. *Cladobotryum protrusum* is the first fungus genome to be sequenced as the pathogenic fungus causing cobweb disease [24]. The analysis confirmed that the fungus belongs to the family Hypocreaceae, and genes from CAZymes, secondary metabolites, P450, and the pathogen–host interaction database (PHI) all contribute to its mycotrophic lifestyle. In subsequent studies, the genomes of two additional cobweb disease pathogenic fungi, *C. dendroides* and *C. mycophilum*, were sequenced [13,15]. Through genome analysis, one can examine genes that play an important role during the parasitic relationship with mushrooms, such as carbohydrate-active enzymes (CAZymes), cytochrome P450, peptidases, transporters, and genes involved in secondary metabolites. Understanding the pathogens at the molecular level is essential to gain new insights into the mechanisms of disease establishment within the host. To this aim, additional genomic studies are necessary, encompassing a broader array of mushroom fungal pathogens.

In the present study, three cobweb pathogenic fungi from different species were isolated from *A. bisporus*, *F. velutipes*, and *M. importuna*, respectively. The genome of the three fungal strains were sequenced and annotated. The objective of the study was to identify key factors causing cobweb disease through genomic and comparative genomic analysis. The analysis of these strains is also critical for studying and preventing cobweb disease.

## 2. Materials and Methods

### 2.1. Fungal Strains and Genomic DNA Preparation

The three strains, *C. protrusum*, *C. mycophilum*, and *H. aurantius*, used in this study were provided by Tai’an Academy of Agricultural Sciences (Tai’an, China). These strains were identified as the pathogens of cobweb disease that infect fruiting bodies of *M. importuna*, *A. bisporus*, and *F. velutipes*, respectively [18,23,34]. The mycelia of these strains were cultured and maintained on potato dextrose agar (PDA) plates. For genome sequencing, the mycelia were inoculated on the PDA plates overlaid with a cellophane membrane and cultured for 7 days at 25 °C in darkness. Genomic DNAs were extracted using the NucleoBond HMW DNA kit (Macherey–Nagel, Düren, Germany) according to the manufacturer’s instructions. DNA concentration and purification were measured with the Nanodrop One (Thermo Fisher Scientific, Waltham, MA, USA).

### 2.2. Genome Sequencing and Assembly

The extracted genome DNA was sequenced using the technology of Oxford Nanopore and Illumina platforms. The Nanopore library was constructed using the Oxford Nanopore LSK-109 kit (Oxford Nanopore Technologies, Oxford, UK), and the Nanopore library was sequenced on the PromethION platform by Benagen Ltd. (Wuhan, China). After trimming and filtering, in total, clean data of 9.19, 10.75, and 8.18 Gb were generated for *C. protrusum*, *C. mycophilum*, and *H. aurantius*, respectively. Illumina paired-end sequencing was performed on an Illumina HiSeq2500 platform under 150 bp mode in the same company. The raw fastq data were filtered using the fastp software v0.23.2 [35]. After filtration, a total of 6.40, 6.26, and 6.55 Gb of clean data were kept for *C. protrusum*, *C. mycophilum*, and *H. aurantius*, respectively. Kmer (19 bp) was calculated using Jellyfish software v2.2.10, and the genome survey was analyzed using GenomeScope 2.0 (p = 1 and p = 2, m 20,000) [36]. The genome was assembled using Necat software v2020-08-03 with default parameters (GENOME_SIZE = 40,000,000, PREP_OUTPUT_COVERAGE = 100, and CNS_OUTPUT_COVERAGE = 90) [37]. Polishing of the assembled genome was performed in two iterations using Racon v1.5.0 (https://github.com/lbcb-sci/racon; accessed on 12 December 2023) with Nanopore reads and default parameters. The polished scaffolds were further polished with Pilon v1.24 through two iterations using filtered Illumina reads [38].

### 2.3. Gene Prediction and Function Annotation

Augustus software v3.4.0 was used to predict genes with *laccaria_bicolor* models, and *ab initio*-based gene prediction was performed using GeneMark-ES v4.69 with default parameters [39,40]. The two prediction results were integrated using EVidenceModeler software v1.1.1 [41]. The completeness of the gene prediction was evaluated using the BUSCO software v5.1.2 with fungi_odb10. Functional annotation of the three fungi’s predicted protein-coding sequences (CDS) was performed by using Diamond to search against several protein databases such as the eggNOG-mapper, the Pfam database, and the SwissProt database [42,43]. Circular layouts were generated using Circos software v0.69 (http://circos.ca/; accessed on 20 December 2022) [44].

### 2.4. Comparative Genomic Analysis

Pairwise average nucleotide identity (ANI) was calculated using fastANI software v1.33 [45]. Collinearity analysis of pathogenic fungi (*H. aurantius*, *H. rosellus*, *C. mycophilum*, and *C. protrusum*) causing cobweb disease was conducted using MCScanX software [46]. Gene families and orthogroups were analyzed using the OrthoFinder software v2.5.4 [47]. The phylogenomic tree was constructed by concatenating single-copy orthologous protein sequences, which were then visualized using the FastTree software v2.1 [48]. Divergent time analysis was performed using PAML software packages v4 MCMCtree using reference divergence time between *Lentinula edodes* and *Saccharomyces cerevisiae* (626–806 MYA) and between *Aspergillus niger* and *Neurospora crassa* (233.8–367.0 MYA) obtained from the Timetree database http://timetree.org (accessed on 10 June 2024).

### 2.5. Analysis of Secretory Protein and Pathogenicity-Related Genes

CAZymes were annotated using dbCAN v3.0.2 software with the Hmmer search engine and default parameters [49]. Protein-signal peptides were predicted using SignalP v5.0b software, and transmembrane structures were predicted using TMHM v2.0c [50,51]. Signal-peptide proteins without transmembrane structures are considered secretory proteins. The proteins from PHI were downloaded from https://www.phi-base.org Version 4.17 (released on 1 May 2024; accessed on 20 June 2024) [52]. Proteins from the database of fungal virulence factors (DFVF) were downloaded from http://sysbio.unl.edu/DFVF (released in 2019, with 2058 entries; accessed on 20 June 2024) [53]. Genes in PHI and DFVF were identified using BlastP software v2.2.28 against these databases with an E-value of 0.00001.

## 3. Results and Discussion

### 3.1. Genome Information of Three Cobweb Disease Pathogens

**Figure 1:**
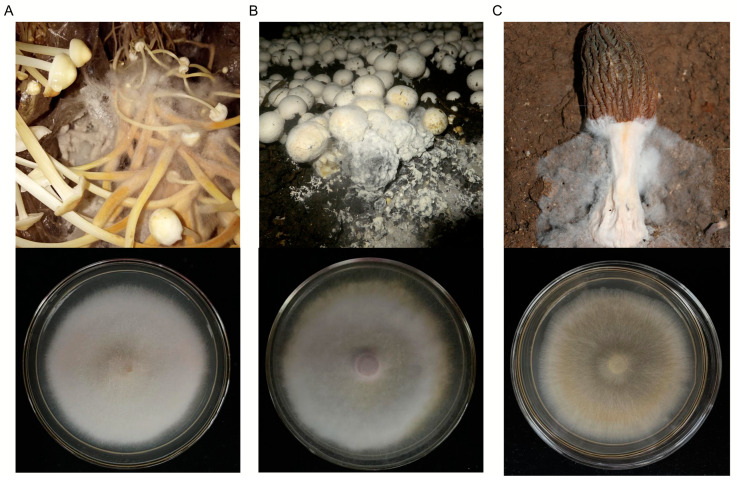
Infected mushrooms and colony morphology of three cobweb disease pathogens used in this study. (**A**) Field symptoms of cobweb disease on *F. velutipes* and colonies of the cobweb disease strain *H. aurantius* CB-Fv on PDA media at 25 °C. (**B**) Field symptoms of cobweb disease on *A. bisporus* and colonies of the cobweb disease strain *C. mycophilum* CB-Ab. (**C**) Field symptoms of cobweb disease on *M. importuna* and colonies of the cobweb disease strain *C. protrusum* CB-Mi.

Three mycoparasites causing cobweb disease were isolated and identified previously. *Hypomyces aurantius* CB-Fv was isolated from *F. velutipes* [18], *C. mycophilum* CB-Ab was isolated from a white button mushroom (*A. bisporus*) [34], and *C. protrusum* CB-Mi was isolated from cultivated *M. importuna* [23] (Figure 1).

**Figure 2:**
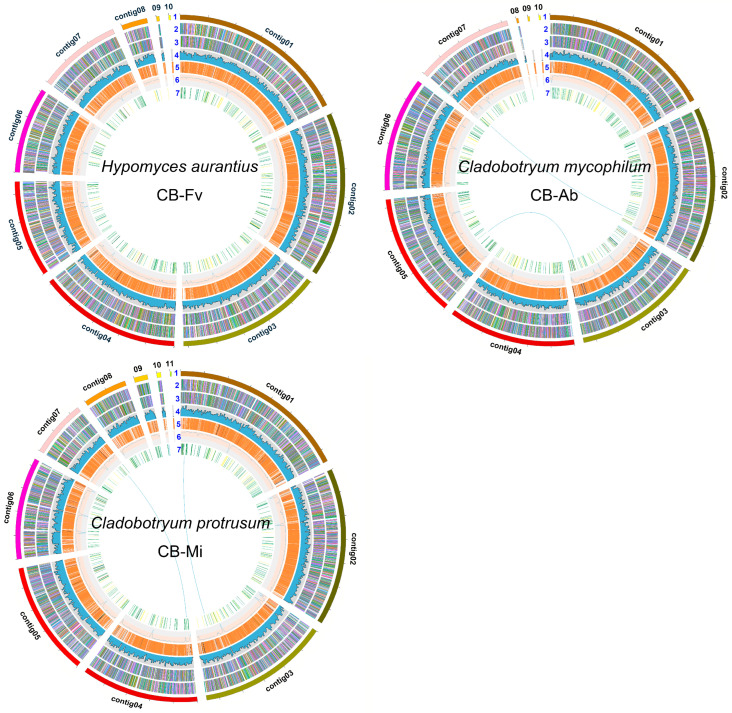
Genome maps of three cobweb disease pathogens *H. aurantius* CB-Fv, *C. mycophilum* CB-Ab, and *C. protrusum* CB-Mi. Layer 1, contigs; Layers 2 and 3, predicted genes in forward and reverse strand; Layer 4, gene density; Layer 5, repeat sequences; Layer 6, GC content; Layer 7, CAZymes. Links within and between contigs represent collinear blocks generated from MCScanX. The plot was visualized using Circos software with a window size of 50 kb.

The pathogenicity of the three strains for cobweb disease was confirmed according to Koch’s postulate previously. Here, the genome sequences of the three strains were sequenced using Oxford Nanopore and Illumina sequencing platforms. As shown in Supplementary Figure S1, a genome survey by GenomeScope was used to evaluate the heterozygosity of the three strains. The heterozygous rate was 0.073%, 0.128%, and 0.101% for *H. aurantius*, *C. mycophilum*, and *C. protrusum*, respectively. These results confirmed the homokaryotic nature of the three strains. The de novo assembly of the *H. aurantius* genome yielded 33.19 Mb, consisting of 10 contigs with a contig N50 length of 5.18 Mb and a contig N90 length of 2.99 Mb. The genome size of *C. mycophilum* was 39.83 Mb, consisting of 10 contigs with a contig N50 length of 5.50 Mb and a contig N90 length of 4.24 Mb. The *C. protrusum* genome contains a total length of 38.10 Mb, containing 10 contigs with a contig N50 length of 5.55 Mb and a contig N90 length of 2.41 Mb (Table 1, Figure 2). The genomes of the two *Cladobotryum* pathogens sequenced in this study exhibited similar genome sizes and GC content compared to those previously reported for the *Cladobotryum* species (Supplementary Table S1). This also suggested the completeness of the genomes sequenced in this study. The genome size of *H. aurantius* was smaller than that of other reported cobweb disease pathogens from the genus *Cladobotryum* (>36 Mb), and it was also smaller than the genomes of the other two sequenced strains from *Hypomyces* genus (>38.48 Mb) (Supplementary Table S1). In addition, the GC content of the *H. aurantius* genome was the highest among these reported strains. *Hypomyces aurantius* represents the first cobweb pathogen from the *Hypomyces* genus to be sequenced, providing valuable information for gaining deeper insights into cobweb disease pathogens. Most fungal diseases are attributed to filamentous fungi, primarily due to the similarity in their nutritional requirements to those of edible mushrooms. These fungi can secrete numerous proteins, allowing them to parasitize edible mushrooms by outcompeting for nutrients and through direct invasion. The genomes of these fungi generally range from 35 to 45 Mb, likely owing to the requirement to encode an extensive array of CAZymes and other pathogenic genes [54,55,56,57]. In contrast, yeast-like pathogenic fungi, such as *Saccharomycopsis phalluae,* possess a genome size of merely 14 Mb. Analyses of published filamentous fungal genomes indicate that various pathogenic fungi exhibit similar potential pathogenic mechanisms [58].

### 3.2. Genome Annotation

The protein-encoding genes in the assembled genomic sequence were predicted based on sequence homology and de novo gene predictions. Gene models of 9791, 11,365, and 113,038 were predicted with a total length of 15.66 Mbp (47.2% of the genome), 18.11 Mbp (45.5% of the genome), and 17.86 Mbp (46.9% of the genome) for *H. aurantius*, *C. mycophilum*, and *C. protrusum* (Figure 2, Table 2). The predicted genes in *H. aurantius*, *C. mycophilum*, and *C. protrusum* genomes showed an average length of 1590.3 bp, 1593.5 bp, and 1618.1 bp, an average exon length of 554.7 bp, 561.1 bp, and 572.9 bp, an average intron length of 83.6 bp, 88.5 bp, and 83.1 bp, and an average intron number of 1.87, 1.84, and 1.82, respectively (Table 2). The completeness of assemblies was evaluated using BUSCO v5.1.2 (fungi_odb10, ascomycota_odb10, sordariomycetes_odb10) [59]. The results of BUSCO analysis of *H. aurantius* CB-Fv, *C. mycophilum* CB-Ab, and *C. protrusum* CB-Mi based on sordariomycetes_odb10 were 98.2%, 98.4%, and 98.8%, respectively. The high BUSCO completeness indicated the quality of the genome assembly and high fidelity of gene prediction.

Functional annotations of the predicted genes were performed using publicly available databases of Swissprot, GO, KEGG, eggNOG, and Pfam. Of the identified genes, the largest number of genes was annotated by the eggNOG database, and the least number of genes was annotated by GO analysis (Supplementary Table S2). There were 5843, 6580, and 6511 genes of *H. aurantius*, *C. mycophilum*, and *C. protrusum* annotated in the SwissProt database, accounting for 59.68%, 57.90%, and 58.99% of the total number of genes. Based on the similarity of protein domains, 7278 (74.33%), 8390 (73.82%), and 8214 (74.42%) genes of *H. aurantius*, *C. mycophilum*, and *C. protrusum* were annotated by Pfam database. There were 3951 (40.35%), 4148 (36.50%), and 4092 (37.07%) genes of *H. aurantius*, *C. mycophilum*, and *C. protrusum* annotated by the KEGG pathway, of which most genes involved in metabolism (Supplementary Table S2).

### 3.3. Evolution and Comparative Genomic Analysis

**Figure 3:**
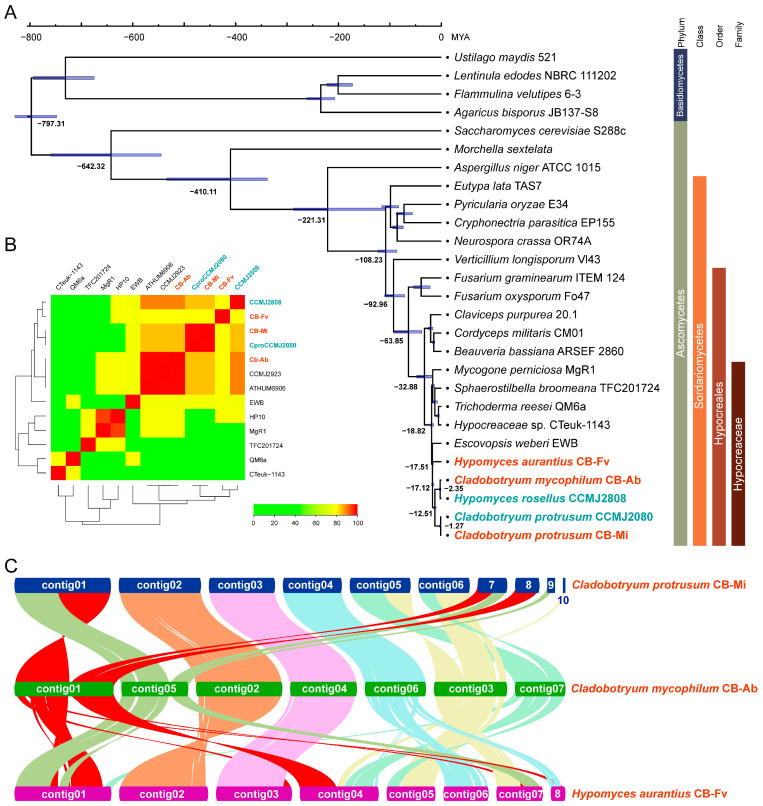
Evolutionary and comparative genomic analysis of three cobweb disease strains. (**A**) Fungi phylogenetic tree inferred from 101 single-copy orthologs among 27 fungi species identified using OrthoFrinder software. (**B**) The cluster heatmap of ANI values between 13 strains and the specific value were listed in Supplementary Table S3. (**C**) Genome collinearity among three cobweb disease strains, *H. aurantius* Cb-Fv, *C. mycophilum* CB-Ab, and *C. protrusum* CB-Mi. Each line connects a pair of collinearity blocks between two genomes.

Both *Cladobotryum* and *Hypomyces* come from the family Hypocreaceae. Genomes of seven genera, including *Cladobotryum* and *Hypomyces*, of the Hypocreaceae family have been published in the NCBI database (May 2024). The comparison of all genomes from *Cladobotryum* and *Hypomyces* and the representative strains from the other five genera were performed using fastANI software. While the ANI value is typically employed to determine species boundaries of prokaryotic genomes [45], the software was simply used to present the genome similarities between different fungal genomes in this study. Figure 3 reveals that strains from the same species showed high ANI values (>98%) with each other. *Cladobotryum protrusum* strain CB-Mi shared the highest ANI value of 98.96% with *C. protrusum* strain CCMJ2080, also a cobweb pathogen isolated from *C. comatus*, while the ANI values with other fungi were all lower than 85%. Strain CB-Ab showed the highest ANI values of 98.49% and 98.95% with strain ATHUM6906 and strain CCMJ2923, respectively, while ANI values with other strains were all below 90%. Strain CB-Fv, the first sequenced strain of *H. aurantius*, exhibited its highest ANI value of 79.59% with *H. perniciosus* HP10. Thus, ANI analysis corroborates taxonomic classifications to some extent, reflecting the evolutionary relationships among different strains.

To further investigate the evolutionary relationships of the three fungi and the divergent time from a common ancestor, phylogenetic analyses were conducted between the three fungi and 24 other fungi species (20 Ascomycetes and 4 Basidiomycetes). A total of 101 single-copy orthologous genes were identified using OrthoFinder and used for phylogenetic reconstruction, and species divergence time was estimated based on amino-acids sequences using mcmctree (Figure 3A). The results indicated that strains from the Sordariomycetes class cluster together. The genera *Hypomyces* and *Escovopsis* diverged 17.51 million years ago. Based on ANI analysis and genomic phylogenetic analysis, strain CCMJ2808 should belong to the genus *Cladobotryum*. The genera *Hypomyces* and *Cladobotryum* separated 17.12 million years ago. This suggests that cobweb disease pathogens share genetically similar, indicating a common infection mechanism at the genetic level.

Collinearity analysis of the three strains revealed a high degree of synteny conservation, as illustrated in Figure 3C: Contig02 and contig04 of strain CB-Ab were highly conserved, exhibiting almost no chromosomal recombination events among the three strains. Portions of contig01 and contig05 of strain CB-Ab displayed collinearity with contig01 of strains CB-Mi and contig01 of strain CB-Fv, whereas the remaining parts of the two contigs are dispersed across different locations in the other two strain genomes. Contig06 of strain CB-AB showed high collinearity with contig04 of strain CB-Mi but was fragmented across different positions in the genome of strain CB-Fv. Contigs03 and contig07 of strain CB-Ab exhibited numerous fragmentation events compared to the other two genomes. Overall, fewer genomic rearrangement events occurred between strains CB-Mi and CB-Ab compared to CB-Fv and the other two strains, which was consistent with the previous phylogenetic analysis. The results indicated that there are conserved contigs and variable contigs at the chromosomal level, with more rearrangement events occurring in the non-conserved variable contigs.

### 3.4. Comparative Analysis of CAZymes

**Figure 4:**
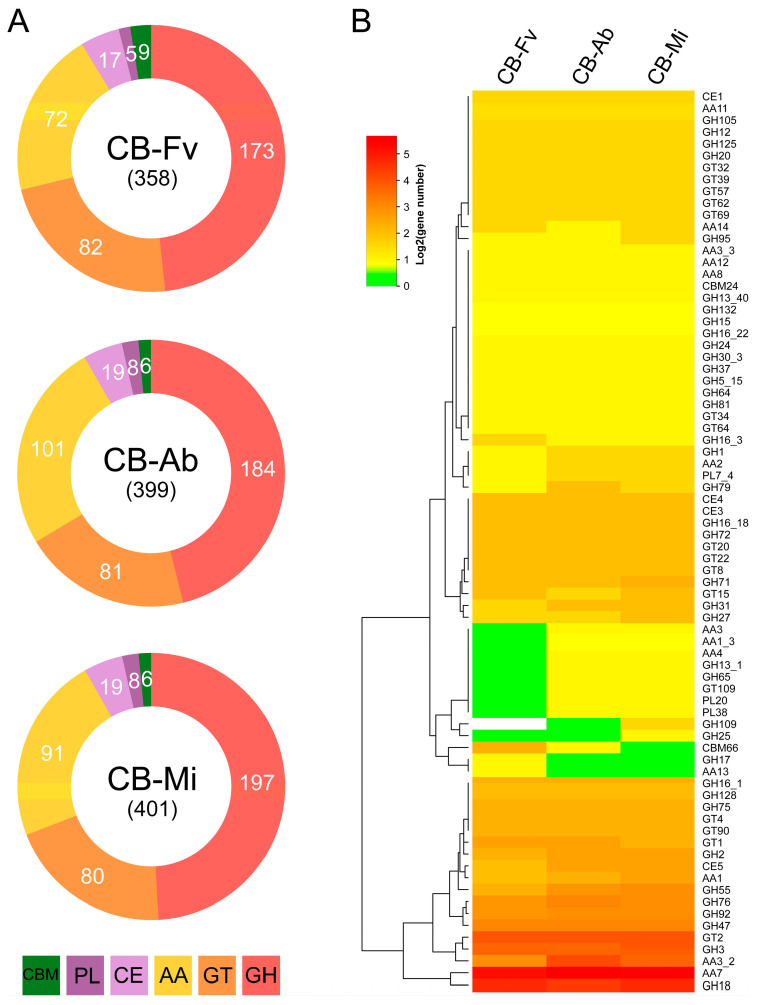
Distribution of CAZymes genes in three cobweb pathogens. (**A**) Distribution of CAZymes genes in *H. aurantius* Cb-Fv, *C. mycophilum* CB-Ab, and *C. protrusum* CB-Mi. (**B**) Heatmap representing the gene number (>1) of CAZyme families in three strains. Abbreviations: GH: glycoside hydrolase, GT: glycosyl transferases, CE: carbohydrate esterases, AA: auxiliary activities, CBM: carbohydrate-binding molecules, PL: polysaccharide lyases.

The genome of *H. aurantius* CB-Fv, *C. mycophilum* CB-Afb, and *C. protrusum* CB-Mi contained 358, 399, and 401 CAZymes with a high diversity of families, including a total of six classes: glycoside hydrolases (GHs = 173, 184, and 197), glycosyl transferases (GTs = 82, 81, and 80), carbohydrate esterases (CEs = 17, 19, and 19), auxiliary activities (AAs = 72, 101, and 91), carbohydrate-binding molecules (CBMs = 9, 6, and 6), and polysaccharide lyases (PLs = 5, 8, and 8) (Figure 4A, Supplementary Tables S4–S6). The family with the highest number of genes was the AA7 family. The three strains, *H. aurantius* CB-Fv, *C. mycophilum* CB-Ab, and *C. protrusum* CB-Mi, had 36, 51, and 45 genes, respectively, in this family. Genes in this family are primarily involved in the synthesis of secondary metabolites, such as the antifungal substance trichoxide, the defense-related metabolite zearalenone, and chanoclavine [60,61]. Cell wall-degrading enzymes have been reported to be associated with mycoparasitism. The white mildew disease fungus *Calcarisporium cordycipiticola* could degrade the hyphase of its host *Cordyceps militaris* [57]. Genes from the GH18 family numbers were 27, 22, and 27 in the three pathogens, respectively, second only to the AA7 family (Figure 4B, Supplementary Tables S4–S6). The enzymes of this family were mainly annotated as endochitinases and chitotriosidases. These enzymes can hydrolyze the β-(1, 4)-linkage between adjacent N-acetyl glucosamine residues of chitin, playing a pivotal role in the context of infectious diseases [62]. The induction of chitinolytic enzymes were observed in the interaction between *Trichoderma harzianum* and *Sclerotium rolfsii* [63]. A transcriptome analysis of the wet bubble disease *Mycogone perniciosa* revealed that several GH18 chitinases were significantly upregulated during the infection process targeting *A. bisporus* [64]. Calonje et al. [65] reported that hydrolytic enzymes, including chitinases, were produced by *Verticillium fungicola*, a dry bubble pathogen, in cultures with *A. bisporus* vegetative mycelial cell walls as the only carbon source. Similar results were also reported for the green mould disease fungus *Trichoderma aggressivum* [66]. The results were consistent with our genomic analysis results, indicating that genes in the GH18 family play a crucial role in cobweb disease pathogenicity [67]. Another gene family, GH55, mainly consists of glucan endo-1,3-beta-glucosidase, which are also pathogenesis-related genes involved in the degradation of beta-1,3-glucan in host cell walls [68]. The enzyme activity of 1,3-beta-glucanase was also detected in the culture of *V. fungicola* with *A. bisporus* myvelial cell walls as the substrate [65]. The three pathogens had five, seven, and eight genes, respectively, in the GH55 family, which is also higher than the number in *F. velutipes* (3), *A. bisporus* (1), and *P. ostreatus* (2), suggesting a possible link to their pathogenicity. Overall, genomic analysis confirmed that cell wall-degrading enzymes play key roles in the infection of mushrooms.

### 3.5. Pathogenicity-Related Genes

**Figure 5:**
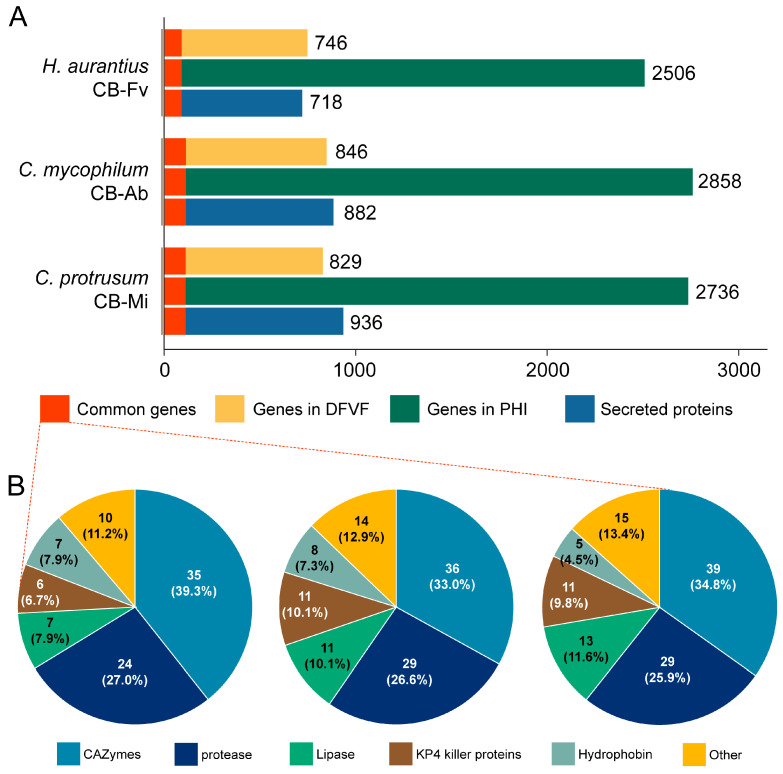
Distribution of pathogenicity-related genes in three cobweb disease strains. (**A**) The distribution of secreted proteins, genes identified in the PHI database, and genes identified in the DFVF database in *H. aurantius* Cb-Fv, *C. mycophilum* CB-Ab, and *C. protrusum* CB-Mi, respectively. Common genes were genes that belong to all these categories. (**B**) The gene distribution in common genes of the three cobweb disease strains.

As secreted proteins are critical virulence determinants in fungal pathogens, we compared the secreted proteins of three strains to gain information about their composition and conservation. A total of 717, 882, and 936 secreted proteins (with signal peptide and without transmembrane structure) were predicted in the genomes of strain CB-Fv, CB-Ab, and CB-Mi, respectively (Figure 5A, Supplementary Tables S4–S6). To identify potentially pathogenic genes in these cobweb disease fungi, the whole genomes were analyzed via BLASTP against the PHI and DFVF databases, which predict protein function during host infection. There were 2506, 2758, and 2736 genes annotated in the PHI database for *H. aurantius* CB-Fv, *C. mycophilum* CB-Ab, and *C. protrusum* CB-Mi, respectively, based on sequence identity ≧ 30% and alignment coverage ≥ 50% (Figure 5A, Supplementary Tables S4–S6). The DFVF database is an inclusive database of recognized fungal virulence factors. It collects 2058 pathogenic genes released by 228 fungal strains from 85 genera. Currently, there were 746, 846, and 829 genes identified in the genomes of *H. aurantius*, *C. mycophilum*, and *C. protrusum*, respectively (sequence identity ≥ 30% and alignment coverage ≥ 50%), against the DFVF database (Figure 5A, Supplementary Tables S4–S6). In the genomes of *H. aurantius* CB-Fv, *C. mycophilum* CB-Ab, and *C. protrusum* CB-Mi, 89, 109, and 112 secreted proteins (common genes) were identified in both the PHI and DFVF databases, respectively. Among the common genes, the majority of proteins (>90%) of each strain had homologs in the common genes of the other two strains, indicating that these proteins (core genes) were essential to the pathogenicity of the three web blight pathogens. Some of the core genes were CAZymes, including the previously mentioned chitinases and glucanases, while most of the other genes were annotated as proteases (peptidases), lipases, hydrophobins, KP4 killer proteins, and ROS-related enzymes (Figure 5B, Supplementary Tables S4–S6). The induction of a laccase gene, lcc2, by *A. bisporus* can provide defense against *T. aggressivum*, a green mould disease. The expression of proteases might selectively degrade the defensive enzymes secreted by the host mushroom [69]. The upregulated expression of a proteinase gene, *prb1*, was detected when *T. aggressivum* was co-cultured with *A. bisporus* [66]. The results suggested that these genes have roles in pathogenicity. Lu’s study revealed that ROS levels increased when *Phallus rubrovolvatus* was co-cultured with a pathogenic fungus *Trichoderma koningiopsis* [70]. Therefore, ROS-related enzymes in cobweb disease may be associated with stress defense against host mushrooms.

Based on the analysis of genome, we can infer that the cobweb disease pathogens may inhibit the growth of host cells by secreting antifungal metabolites (compounds synthesized by enzymes from the AAs family and KP4 killer proteins [71]). Subsequently, they secrete chitinases, glucan endo-1,3-beta-glucosidases, and other enzymes (such as proteases and lipases) to degrade the host cell walls, providing nutrients for their own growth and, thus, completing the infection process. Additionally, hydrophobins are deeply involved in and regulate the infection processes. In future research, it will be essential to investigate the specific secreted proteins and CAZymes related to pathogenicity through transcriptomics or proteomics.

## 4. Conclusions

In summary, high-quality genomes of three cobweb disease fungi, *H. aurantius* Cb-Fv, *C. mycophilum* CB-Ab, and *C. protrusum* CB-Mi, were sequenced using the Illumina and Nanopore platforms. Genomic and comparative analysis indicate that the pathogens of cobweb disease might inhibit the growth of host mycelia by secreting antifungal substances and cell wall-degrading enzymes to hydrolyze the host mycelia, thereby completing the infection process. The in-depth genomic information generated in this study will contribute to the control of cobweb disease in mushroom production.

## Figures and Tables

**Table 1:** De novo genome assembly and features of *H. aurantius*, *C. mycophilum*, and *C. protrusum*.

	*H. aurantius* CB-Fv	*C. mycophilum* CB-Ab	*C. protrusum* CB-Mi
Genome size	33.19 Mb	39.83 Mb	38.10 Mb
Total contig number	10	10	11
Longest contig	6.38 Mb	7.44 Mb	7.53 Mb
Contig N50	5.18 Mb	5.50 Mb	5.55 Mb
Genome size	33.19 Mb	39.83 Mb	38.10 Mb
Contig N90	2.99 Mb	4.24 Mb	2.41 Mb
GC content	49.00%	48.00%	48.00%
Sequencing platform	Napopore, Illumina	Napopore, Illumina	Napopore, Illumina
Isolation host	*F. velutipes*	*A. bisporus*	*M. importuna*

**Table 2:** Characteristics of the gene prediction of *H. aurantius*, *C. mycophilum*, and *C. protrusum*.

	*H. aurantius* CB-Fv	*C. mycophilum* CB-Ab	*C. protrusum* CB-Mi
Gene number	9791	11,365	11,038
Total length	15.57 Mb	18.11 Mb	17.86 Mb
Average length	1590.3 bp	1593.1 bp	1618.1 bp
Average exon length	554.7 bp	561.1 bp	572.9 bp
Average exon number	2.87	2.84	2.82
Average Intron length	83.6 bp	88.5 bp	83.1 bp
Average intron number	1.87	1.84	1.82

## Data Availability

The original contributions presented in the study are included in the article/Supplementary Material, further inquiries can be directed to the corresponding authors or at https://file.mushroomlab.cn.

## Supplementary Materials

The following supporting information can be downloaded at: https://www.mdpi.com/article/10.3390/foods13172779/s1, Figure S1: Genome survey results generated by Genome Scope software; Figure S2: BUSCO analysis results of three cobweb disease fungi; Table S1: Characteristics of genomes from *Hypomyces* and *Cladobotryum* genera; Table S2: Predicted gene numbers identified in eggNOG, SwissProt, Pfam, KEGG, and GO databases in three cobweb disease strains; Table S3: ANI values between the genome of different fungi. The ANI values were generated using fastANI software; Table S4: Pathogenicity-related genes in *Hypomyces aurantius* Cb-Fv; Table S5: Pathogenicity-related genes in *Cladobotryum mycophilum* CB-Ab; Table S6: Pathogenicity-related genes in *Cladobotryum protrusum* CB-Mi.

## Abbreviation

*Agaricus bisporus* (*A. bisporus*); *Coprinus comatus* (*C. comatus*); *Morchella importuna* (*M. importuna*); *Oudemansiella raphanipes* (*O. raphanipes*); *Lentinula edodes* (*L. edodes*); *Pleurotus ostreatus* (*P. ostreatus*); *Pleurotus pulmonarius* (*P. pulmonarius*); *Pleurotus eryngii* (*P. eryngii*); *Flammulina velutipes* (*F. velutipes*); *Hypsizygus marmoreus* (*H. marmoreus*); *Cladobotryum mycophilum* (*C. mycophilum*); *Hypomyces odoratus* (*H. odoratus*); *Morchella sextelata* (*M. sextelata*); *Cladobotryum dendroides* (*C. dendroides*); *Cladobotryum protrusum* (*C. protrusum*); *Cladobotryum varium* (*C. varium*); *Auricularia cornea* (*A. cornea*); *Auricularia heimuer* (*A. heimuer*); *Hypomyces aurantius* (*H. aurantius*); *Hypomyces mycophilus* (*H. mycophilus*); *Hypomyces cornea* (*H.s cornea*); *Hypomyces rosellus* (*H. rosellus*); *Trichoderma aggressivum* (*T*. *aggressivum*); carbohydrate-active enzymes (CAZymes); potato dextrose agar (PDA); coding sequences (CDSs); average nucleotide identity (ANI); pathogen-host interaction database (PHI); database of fungal virulence factors (DFVF); glycoside hydrolase (GH); glycosyl transferase (GT); carbohydrate esterase (CE); auxiliary activity (AA); carbohydrate-binding molecule (CBM); polysaccharide lyase (PL).
