# Supplementary material for: Genome Sequencing of Three Pathogenic Fungi Provides Insights into the Evolution and Pathogenic Mechanisms of the Cobweb Disease on Cultivated Mushrooms
Source: Foods. 2024 Aug 30;13(17):2779. doi: 10.3390/foods13172779 (PMC11394773; doi:10.3390/foods13172779)
Supplement: Supplementary file 1 [file foods-13-02779-s001.zip › Supplementary Materials.pdf]

Supplementary materials

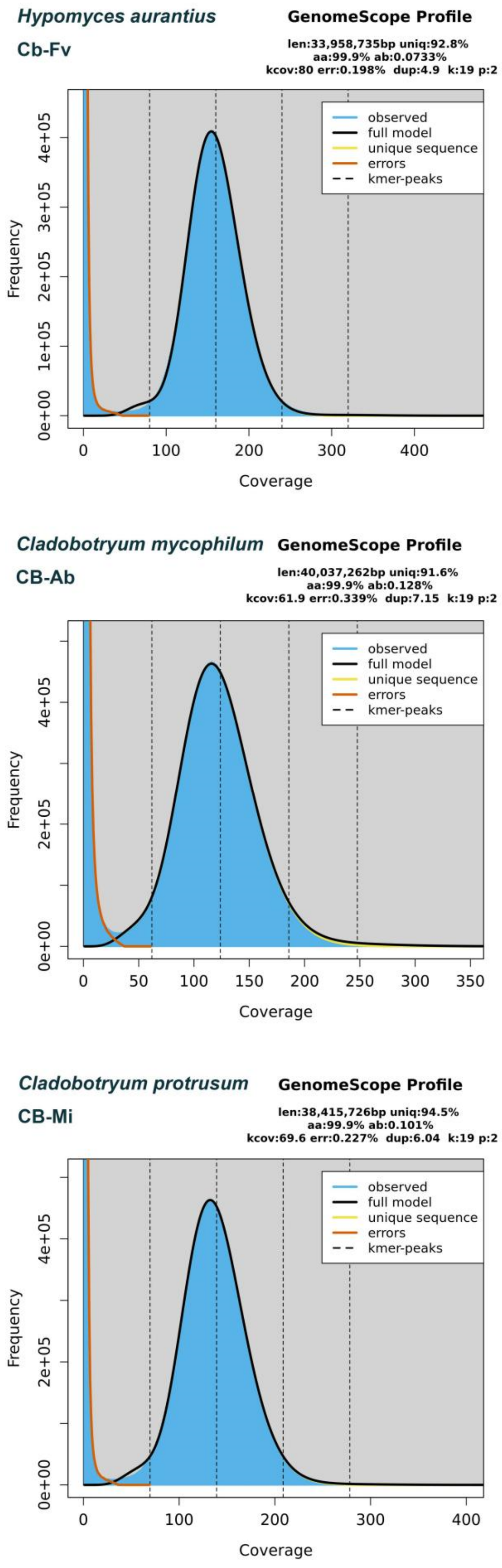

Figure S1. Genome survey results generated by Genome Scope software.

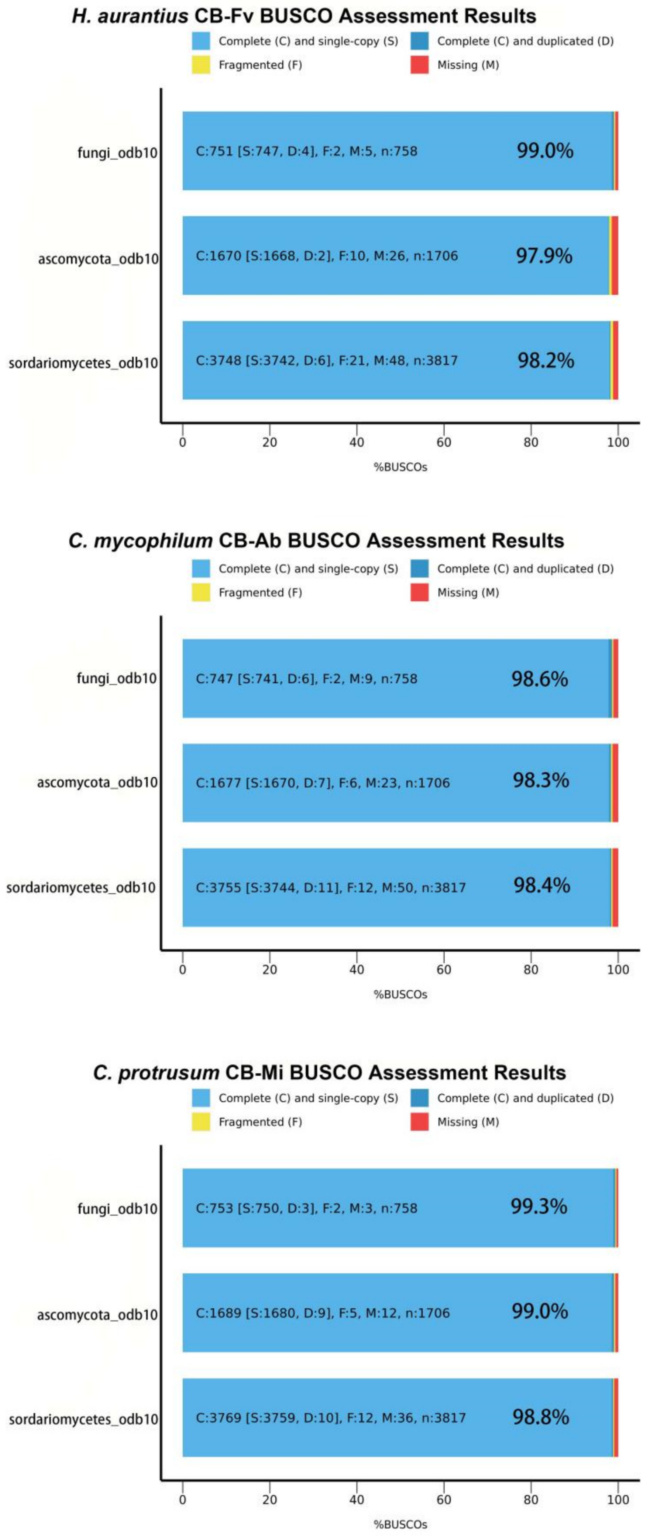

Figure S2. BUSCO analysis results of three cobweb disease fungi.
